# The upregulation of stromal antigen 3 expression suppresses the phenotypic hallmarks of hepatocellular carcinoma through the Smad3-CDK4/CDK6-cyclin D1 and CXCR4/RhoA pathways

**DOI:** 10.1186/s12876-022-02400-z

**Published:** 2022-08-08

**Authors:** Menglin Zhao, Yanyan Wang, Yue Zhang, Xinwei Li, Jiaqi Mi, Qiang Wang, Zhijun Geng, Lugen Zuo, Xue Song, Sitang Ge, Zining Zhang, Mingyue Tang, Huiyuan Li, Zishu Wang, Chenchen Jiang, Fang Su

**Affiliations:** 1 Department of Medical Oncology, The First Affiliated Hospital of Bengbu Medical College, No. 287 Changhuai Road, Bengbu, 233030 Anhui China; 2 Department of Network Information Center, Bengbu Medical College, No. 2600 Donghai Road, Bengbu, 233030 Anhui China; 3 Department of Central Laboratory, The First Affiliated Hospital of Bengbu Medical College, Bengbu, China; 4 Department of Gastrointestinal Surgery, The First Affiliated Hospital of Bengbu Medical College, No. 287 Changhuai Road, Bengbu, 233030 Anhui China; 5 Department of Clinical Medicine Science, Bengbu Medical College, No. 2600 Donghai Road, Bengbu, 233030 Anhui China; 6 Cancer Neurobiology Group, School of Medicine & Public Health, The University of Newcastle, Callaghan, NSW 2308 Australia

**Keywords:** Stromal antigen 3, Hepatocellular carcinoma, Proliferation, Migration, Invasion, Apoptosis, Cell cycle

## Abstract

**Background:**

The stromal antigen 3 (STAG3) gene encodes an adhesion complex subunit that can regulate sister chromatid cohesion during cell division. Chromosome instability caused by STAG3 gene mutation may potentially promote tumor progression, but the effect of STAG3 on hepatocellular carcinoma (HCC) and the related molecular mechanism are not reported in the literature. The mechanism of the occurrence and development of HCC is not adequately understood. Therefore, the biological role of STAG3 in HCC remains to be studied, and whether STAG3 might be a sensitive therapeutic target in HCC remains to be determined.

**Methods:**

The expression and clinical significance of STAG3 in HCC tissues and cell lines were determined by RT–qPCR and immunohistochemistry analyses. The biological functions of STAG3 in HCC were determined through in vitro and in vivo cell function tests. The molecular mechanism of STAG3 in HCC cells was then investigated by western blot assay.

**Results:**

The mRNA expression of STAG3 was lower in most HCC cells than in normal cells. Subsequently, an immunohistochemical analysis of STAG3 was performed with 126 samples, and lower STAG3 expression was associated with worse overall survival in HCC patients. Moreover, cytofunctional tests revealed that the lentivirus-mediated overexpression of STAG3 in HCC cells inhibited cell proliferation, migration, and invasion; promoted apoptosis; induced G1/S phase arrest in vitro; and inhibited tumor growth in vivo. Furthermore, studies of the molecular mechanism suggested that the overexpression of STAG3 increased Smad3 expression and decreased CDK4, CDK6, cyclin D1, CXCR4 and RhoA expression.

**Conclusion:**

STAG3 exhibits anticancer effects against HCC, and these effects involve the Smad3-CDK4/CDK6-cyclin D1 and CXCR4/RhoA pathways. STAG3 is a tumor-suppressor gene that may serve as a potential target for molecular therapy, which provides a new idea for the treatment of HCC.

**Supplementary Information:**

The online version contains supplementary material available at 10.1186/s12876-022-02400-z.

## Introduction

Liver cancer is a malignant tumor with high incidence and mortality rates [1]. According to the 2020 GLOBOCAN database estimate, primary liver cancer was the sixth most commonly diagnosed cancer and the third leading cause of cancer death worldwide in 2020 [2]. In the United States, the incidence of liver cancer increased from 2% in 2007 to 3% in 2016 [3]. In China, liver cancer ranks fourth among men in terms of incidence and third in terms of mortality [4]. Hepatocellular carcinoma (HCC) accounts for 90% of primary liver cancer cases [5]. Despite advances in treatment, the clinical prognosis of patients with advanced HCC remains poor [6]. Because the mechanism of HCC occurrence and development is not clearly understood, exploring this mechanism could provide new ideas and insights for the treatment of HCC.

Stromal antigen (STAG) is composed of stromal antigen proteins (STAG1 2 or 3), chromosomal proteins (SMC3/SMC1α or SMC3/SMC1β) and α45klesin subunits (RAD21/RAD21L or RAD21/REC8) [7]. As a subunit of the specific adhesin complex, the stromal antigen 3 (STAG3) gene is located on chromosome 7q22, and its protein structure contains a C-terminal domain, a 28-amino acid conserved domain and an 86-amino acid domain [8]. The protein encoded by this gene is expressed in the nucleus and can regulate the cohesion of sister chromatids during cell division [9]. Studies on the abnormal expression or mutation of the STAG3 gene in humans have typically focused on follicular development, infertility, premature ovarian failure, spermatogenesis disorders and male infertility [10]. Studies on the function of the STAG3 gene in mice have mainly focused on chromosomal abnormalities and infertility caused by homozygous mutation of STAG3 [11, 12]. A comparative analysis of ovarian tumors identified a SNP in STAG3, and the rs1637001 allele showed loss of specific allele heterozygosity. The data indicate that the STAG3 gene is involved in the development of epithelial ovarian cancer [13]. KALEJS found that STAG3 is abnormally activated in p53-mutated lymphoma and leads to abnormal mitosis and polyploid tumor cells [14]. Another researcher revealed that STAG3 gene mutation in colorectal cancer causes sister chromatid cohesion defects and lead to chromosome instability, which is related to the occurrence of the disease [15]. STAG3 has seldom been reported in tumors, and few studies have investigated the molecular mechanisms underlying tumorigenesis and development. No previous studies have investigated STAG3 in liver cancer. This study aims to provide a direction for research on the molecular mechanism of HCC.

The present study revealed that the expression of STAG3 in most HCC tissues and cell lines was lower than that in paracancerous tissues. In HCC, low expression of STAG3 was associated with increased tumor size and poor prognosis. Furthermore, the overexpression of STAG3 significantly inhibited the proliferation, metastasis and invasion of HCC cells, induced the arrest of HCC cells at the G1/S phase and promoted apoptosis. In addition, this study showed that the overexpression of STAG3 acted as a regulator of HCC progression mainly through regulation of the cell cycle pathway.

## Materials and methods

### Patients and tissue samples

HCC tissue and adjacent tissue specimens from patients seen at the First Affiliated Hospital of Bengbu Medical College Hospital were obtained. The Ethics Committee of Bengbu Medical College [2017] No. 020 approved the study of patient tissue specimens.

### Immunohistochemistry (IHC)

The tissue samples were fixed in 4% paraformaldehyde, embedded in paraffin, sectioned at 4 μm, deparaffinized in different density gradients of dimethylbenzene, and hydrated with ethanol. Antigen retrieval was performed, and the samples were then successively incubated with STAG3 primary antibody (1:150; Abcam, Cambridge, UK) or Ki-67 antibody (1:200; Abcam, Cambridge, UK) overnight at 4 °C, biotin-bound secondary antibody (1:5,000; ZSGB-BIO, Beijing, China) and streptavidin-peroxidase. All then slides were then stained with hematoxylin. An immunohistochemical assessment was performed after the slides were dried and cleared.

### Cell lines and cell culture

A human normal hepatic cell line (MIHA) and hepatoma cell lines (SMMC-7721, Huh-7, BEL-7404, and HLE) were obtained from Biogenetech. All the cells were maintained in Dulbecco’s modified Eagle’s medium (Gibco, Carlsbad, CA, USA) containing 10% fetal bovine serum (Gibco, Carlsbad, CA, USA), 100 μg/ml streptomycin and 100 U/ml penicillin (Solarbio, Beijing, China) at 37 °C in a humidified atmosphere with 5% CO_2_ during the study.

### RT–qPCR

Total RNA was isolated from tissues and cell lines using TRIzol reagent (Ambition, TX, USA) and reverse transcribed into cDNA using a reverse transcription kit (Thermo, MA, USA). The primer sequences were as follows (5′- 3′): STAG3 forward, CCA ATC TTG CGG ATG TAA AGG C, and STAG3 reverse, CGA GTC CTC ATT AAA CTG CTC TG; GAPDH forward, TGA CTT CAA CAG CGA CAC CCA, and GAPDH reverse, CAC CCT GTT GCT GTA GCC AAA. SYBR Green Real-time PCR Master Mix (TOYOBO, Osaka, Japan) was used for amplification. The relative mRNA level of STAG3 was normalized to that of GAPDH. The 2^−ΔΔCt^ method was used to calculate the relative gene expression normalized to that of GAPDH.

### Plasmid construction and cell infection

For construction of the overexpression plasmids of STAG3, GV358 (GeneChem, Shanghai, China) was selected as the vector, and cDNA for STAG3 was obtained from human BEL-7404 and Huh-7 cells and then subjected to PCR amplification with the following specific primers: 5′- GAG GAT CCC CGG GTA CCG GTC GCC ACC ATG TCT TCC CCG TTG CAA AGA GCT GTG G-3′ (forward) and 5′- TCC TTG TAG TCC ATA CCG GTG AAA TCC TCA ATA TCC AGC TCT GTA GAA TC-3′ (reverse).

BEL-7404 and Huh-7 cells were prepared as cell suspensions in serum-free DMEM at a density of 1 × 10^5^ cells/ml. These cells were infected with the STAG3 overexpression lentivirus and its negative control (NC), and the resulting infected cells served as the STAG3-overexpression (OE) and NC groups, respectively. After 6 h of incubation, the residual serum-free DMEM in each well was replaced by DMEM containing 10% FBS. The cells from each group were incubated for 72 h, and the efficiency of infection was examined by western blot analysis.

### Cell counting kit-8 (CCK-8) assay

Cells of the NC and STAG3-OE groups were seeded in 96-well plates and maintained in a humidified incubator at 37 °C with 5% CO_2_ for 1, 2, 3, 4 and 5 days. Ten microliters of CCK-8 (Beyotime, Shanghai, China) solution was added to each well, and the plate was then incubated for 2 h at 37 °C. The absorbance of each well was measured using a microplate reader (Thermo, MA, USA) at a wavelength of 450 nm (OD450 value).

### Scratch wound healing assay

Cells of the NC group and STAG3-OE group were seeded in 6-well plates and cultured to almost 100% confluence. We then scratched a wound in each well with a 20-μl pipette tip. The cells were incubated in culture medium without serum. After 0, 24 and 48 h, images of the cell layer were taken with a microscope (Olympus, Tokyo, Japan). The distance of cell migration at 0, 24 and 48 h after scratching was detected for the assessment of cell motility. The migration rate (%) was calculated as (migrated distance at indicated time)/(initial distance) × 100%.

### Colony formation assay

Cells (2 × 10^3^ per well) of the NC group and STAG3-OE group were seeded in 6-well plates and cultured with complete medium for 1–2 weeks. The colonies were washed, fixed with methanol for 20 min and then stained with a Wright–Giemsa staining kit (Jiancheng, Nanjing, China) following the manufacturer’s instructions. The colonies were washed and counted under a light microscope.

### Flow cytometry

An Annexin V-FITC/PI apoptosis detection kit (Lianke, Hangzhou, China) was used for the detection of cell apoptosis. Cells in the NC and STAG3-OE groups were seeded into 12-well plates and allowed to grow for 6–8 h. The cells were then harvested, washed with cold PBS. Next, resuspended in 400 µl of binding buffer and stained with 5 µl of Annexin-V-FITC in the dark at room temperature for 15 min. The cells were then counterstained with 10 µl of propidium iodide (PI) and incubated in an ice bath in the dark for 5 min.

The cell cycle was assessed using a cell cycle detection kit (Lianke, Hangzhou, China). Cells of the NC and STAG3-OE groups were seeded into 12-well plates and fixed with 70% precooled ethanol overnight at 4 °C. Subsequently, the cells were washed with PBS, centrifuged, resuspended in PBS containing PI and RNase (at a final concentration of 50 µg/ml) and incubated at 37 °C for 30 min. Cell apoptosis and the cell cycle were detected by flow cytometry (BD, NJ, USA).

### Transwell assays

The migration and invasion abilities of cells were assessed by Transwell assays. For the cell invasion assay, precooled medium was added to Transwell chambers, and the upper chamber was covered with Matrigel (Corning, NY, USA). Cells of the NC and STAG3-OE groups were then seeded into the upper chamber and cultured for 24 h. The Transwell chambers were removed, the cells were fixed with precooled methanol, stained and washed, and images were then taken with a microscope. The procedure used for the cell migration assays was similar to that used for the cell invasion assay analysis with the exception that Matrigel coating was not used.

### Western blot assay

Western blot assays were performed to detect the expression of proteins. Protein samples were separated by 10% SDS–PAGE and then transferred to nitrocellulose membranes (Millipore, MA, USA). The samples were subsequently blocked with 5% nonfat milk, and the blots were incubated with specific primary antibodies (1:1000; Abcam, Cambridge, UK) in 5% BSA overnight at 4 °C. Because most of the target proteins have similar molecular weights, only one target protein can be developed by full-length membranes. Therefore, the membranes were cut prior to hybridization with antibodies, and only the region of the target proteins was preserved. After washing with TNET, the nitrocellulose membranes were incubated with HRP-labeled goat anti-rabbit or anti-mouse secondary antibodies (1:5,000; Solarbio, Beijing, China) for 2 h at 37 °C, and specific protein bands were detected by high-sensitive enhanced chemiluminescence (ECL) and visualized using the Tanon Gel Imaging System (Tanon, Shanghai, China). ImageJ software (Software Inquiry, Quebec, Canada) was used to quantify the bands.

### Tumor formation assay

To determine the function of STAG3 in vivo, we established a tumor-bearing mouse model with BEL-7404 (NC) and STAG3-OE BEL-7404 cells. A total of 4 × 10^6^ cells in 200 μl were inoculated subcutaneously into the left back of female BALB/c nude mice (4 weeks, 17–22 g; Charles River, Beijing, China). The temperature was maintained at 22 ± 1 °C, and the humidity was 45–55%. The weights of the nude mice and tumor volumes were measured and recorded every 1 week for up to 4 weeks. The mice were sacrificed after 4 weeks. Tumor tissues were collected and fixed with formalin. All the animal studies were conducted in accordance with the Guide for the Care and Use of Laboratory Animals of the National Institutes of Health and were approved as detailed in Certificate [2017] No. 044.

### Bioinformatics assay

The expression (RNA-sequencing V2) levels of STAG3 in 50 paired samples (HCC tissues and adjacent tissues) were obtained from The Cancer Genome Atlas (TCGA, https://cancergenome.nih.gov/). HCC samples from The Cancer Genome Atlas (TCGA, https://cancergenome.nih.gov/) database were used for classification using R (3.6.3); HCC samples in the project level 3 HTSeq-FPKM RNA-Seq data format from TCGA (https://portal.gdc.cancer.gov/) were also used. A Kaplan–Meier analysis of the overall survival (OS) of HCC patients stratified by STAG3 expression was performed with TIMER2.0, a public resource at http://cistrome.org/TIMER.

### Statistical analysis

The relationships between the expression of STAG3 and clinicopathological features were analyzed by Chi-square tests. Univariate and multivariate analyses were performed using the Cox proportional hazard regression model in a stepwise manner. OS curves were generated using the Kaplan–Meier method and compared by the log-rank test. All data are presented as the means ± SDs. Statistical comparisons of the data were performed by two-tailed or nonpaired Student’s t test. Statistical analyses were performed using SPSS version 22.0 software (IBM Corp., NY, USA). P < 0.05 was considered to indicate statistical significance.

## Results

### STAG3 is downregulated in human HCC tissues and cell lines

**Fig. 1:**
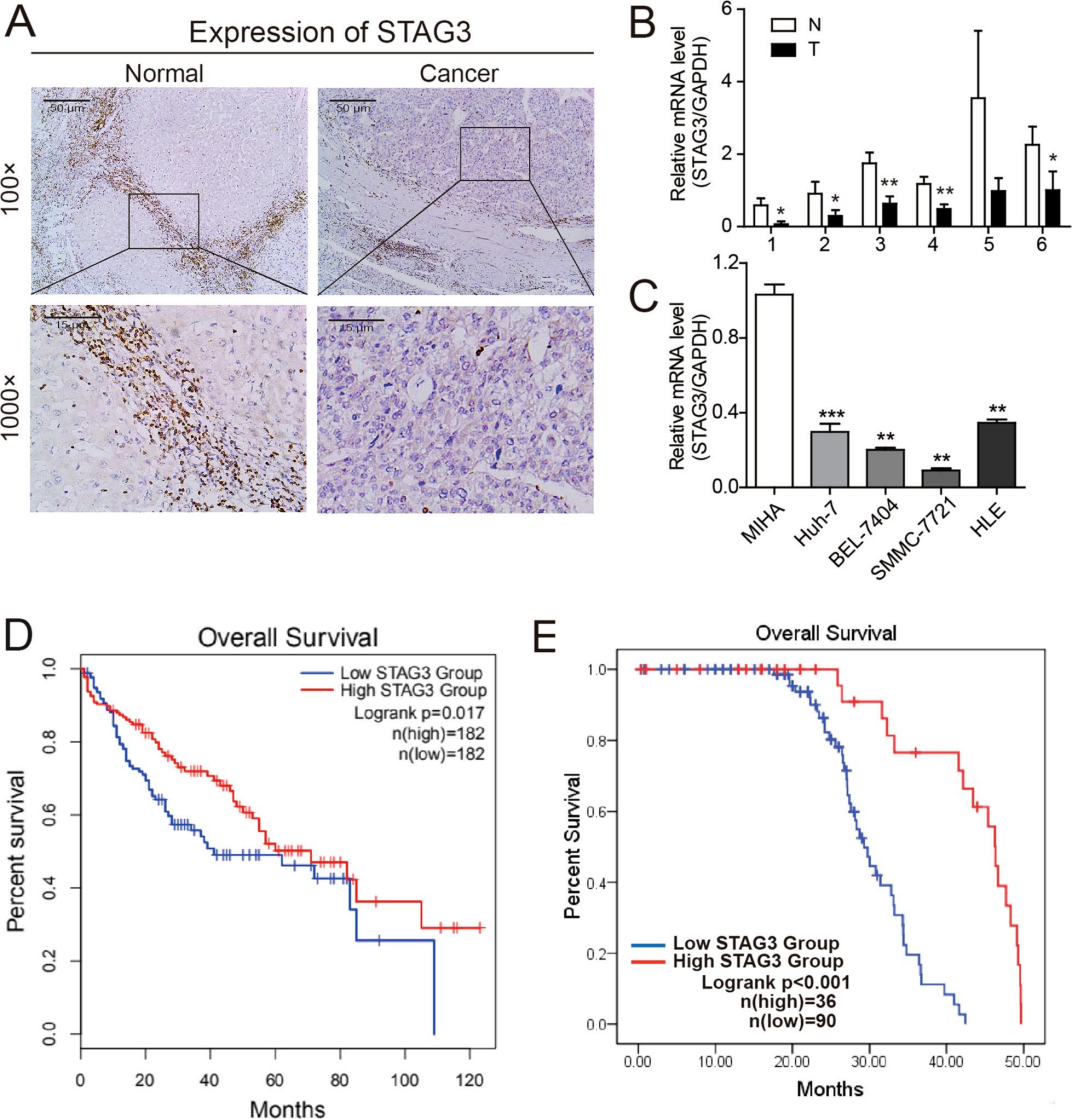
Downregulation of STAG3 expression is frequently observed and associated with poor prognosis in HCC patients. **A** IHC analysis of STAG3 expression in 126 HCC tissue samples and 28 paracarcinoma tissue samples. Representative images are shown (Scale bar, 50 μm, upper picture; scale bar, 15 μm, lower picture). **B** RT–qPCR analysis of the relative mRNA levels of STAG3 in 6 pairs of HCC tumor (T) and nontumor tissues (N). **C** RT–qPCR analysis of STAG3 mRNA expression in HCC cells compared with normal hepatic cells. **D** Patients with low expression levels of STAG3 had a shorter OS than patients with high expression levels of STAG3, as determined using datasets from TCGA. **E** OS analysis of HCC patients based on STAG3 expression in 126 samples. *P < 0.05; **P < 0.01; ***P < 0.001

The analysis of TCGA RNA-sequencing V2 data from 50 paired samples revealed that the mean expression levels of STAG3 were lower in HCC tissues than in paracarcinoma tissues (P < 0.001; Additional file 1: Figure S1). We collected and analyzed 126 samples of HCC tissues and 28 samples of paracarcinoma tissues and found significant differences in STAG3 expression (Fig. 1A). To further assess whether STAG3 was suppressed in HCC, the mRNA expression of STAG3 in six freshly frozen paired HCC and paracarcinoma tissues was evaluated by relative quantification. The results indicated that the mRNA levels of STAG3 were lower in HCC tissues than in adjacent tissues (Fig. 1B). Furthermore, to investigate the expression of STAG3 in HCC, a normal hepatocyte line (MIHA) and HCC cell lines (BEL-7404, Huh-7, SMMC-7721 and HLE) were used for RT–qPCR detection, and the results showed that STAG3 expression was downregulated in HCC cells compared with normal hepatocytes (Fig. 1C). These results demonstrated that STAG3 is expressed at low levels in HCC. Therefore, we speculated that STAG3 may be expressed at low levels and could play an important role in HCC development and progression. See Additional file 2.

### Low STAG3 protein expression is associated with advanced clinical pathological features and a poor prognosis in HCC patients

To explore the role of STAG3 in HCC, we found that the STAG3 protein level was significantly correlated with the T stage, pathological stage and tumor status in the TCGA dataset (Table 1). Furthermore, we evaluated the expression of the STAG3 protein in 126 patients with HCC and analyzed the associations between the STAG3 protein levels and clinicopathological characteristics. The clinical data of all the patients are shown in Table 2, and the results demonstrated that the STAG3 protein levels were significantly correlated with the AFP level (≤ 20/ > 20 ng/ml; P = 0.014), cirrhosis (negative/positive; P = 0.023), the tumor size (≤ 5/ > 5 cm; P = 0.003) and the pathological stage (I-II/III-IV; P = 0.026). However, other clinicopathological features, such as the patient age, sex, hepatitis B virus (HBV) status and intrahepatic metastasis, were not associated with the expression of STAG3. Moreover, the results from a univariate Cox regression analysis revealed that age (≤ 60/ > 60 years; P < 0.001), AFP level (≤ 20/ > 20 ng/ml; P = 0.003), cirrhosis (negative/positive; P < 0.001), tumor size (≤ 5/ > 5 cm; P = 0.001), pathological stage (I-II/III-IV; P < 0.001) and STAG3 protein level (low/high; P < 0.001) were prognostic factors for OS in HCC patients. A multivariate Cox regression analysis showed that the expression of STAG3 (P = 0.001) combined with the pathological stage (P = 0.010) was an independent prognostic factor for OS in HCC patients (Table 3). In addition, the results from a Kaplan − Meier analysis of TCGA HCC cohort demonstrated that HCC patients in the group with low STAG3 expression had a shorter OS than those in the high-expression group (P = 0.017; Fig. 1D). Similarly, a Kaplan − Meier survival analysis revealed that a lower level of STAG3 expression was associated with a shorter OS time in HCC patients (P < 0.001; Fig. 1E).

**Table 1:** Correlations between the expression of STAG3 and clinicopathological features in HCC patients in TCGA dataset

Clinicopathological features	Number, n_max_ = 384	Low expression, n_max_ = 187(%)	High expression, n_max_ = 187(%)	χ2	P value
*Age, y*					
≤ 60	177	87 (23.3)	90 (24.1)	0.069	0.793
> 60	196	99 (26.5)	97 (26.0)		
*Gender*					
Male	253	124 (33.2)	129 (34.5)	0.680	0.410
Female	121	63 (16.8)	58 (15.5)		
*T stage, n (%)*					
T1-T2	278	126 (34.0)	152 (41.0)	9.041	0.003**
T3-T4	93	58 (15.6)	35 (9.4)		
*N stage, n (%)*					
N0	254	134 (51.9)	120 (46.5)	2.221	0.136
N1	4	1 (0.4)	3 (1.2)		
*M stage, n (%)*					
M0	268	145 (53.3)	123 (45.2)	2.589	0.108
M1	4	2 (0.7)	2 (0.7)		
*Pathological stage*					
I–II	260	117 (33.4)	143 (40.9)	8.695	0.003**
III–IV	90	56 (16.0)	34 (9.7)		
*Tumor status, n (%)*					
Tumor free	114	88 (24.8)	114 (32.1)	5.288	0.021*
With tumor	153	89 (25.1)	64 (18)		

*STAG3* stromal antigen 3, *HCC* hepatocellular carcinoma, *TCGA* The Cancer Genome Atlas.

*P < 0.05, **P < 0.01.

**Table 2:** Correlation between the expression of STAG3 and clinicopathological features in 126 HCC patients

Clinicopathological features	Number (n = 126)	Low expression (n = 90)	High expression (n = 36)	χ2	P value
*Age, year*					
≤ 60	69	50	19	0.080	0.777
> 60	57	40	17		
*Gender*					
Male	103	76	27	1.537	0.215
Female	23	14	9		
*AFP, ng/ml*					
≤ 20	52	31	21	6.054	0.014*
> 20	74	59	15		
*Cirrhosis*					
Negative	71	45	26	5.163	0.023*
Positive	55	45	10		
*HBV*					
Negative	12	6	6	2.761	0.097
Positive	111	81	30		
HCV	3	3	0		
*Tumor size, cm*					
≤ 5	65	39	26	8.593	0.003**
> 5	61	51	10		
*Pathological stage*					
I-II	41	24	17	4.950	0.026*
III-IV	85	66	19		
*Intrahepatic metastasis*					
Negative	114	81	33	0.083	0.773
Positive	12	9	3		

STAG3, stromal antigen 3; HCC, hepatocellular carcinoma. *P < 0.05, **P < 0.01

**Table 3:** Univariate and multivariate Cox regression analysis of overall survival in 126 HCC patients

	Overall survival		
Variables	HR	95% CI	P value
*Univariate analysis*			
Age (y, ≤ 60/ > 60)	3.385	1.829–2.264	< 0.001***
Gender	1.197	0.662–1.162	0.552
AFP (ng/ml, ≤ 20/ > 20)	2.378	1.334–4.238	0.003**
Cirrhosis	2.906	1.608–5.251	< 0.001***
HBV	1.132	0.512–2.502	0.760
Tumor size (cm, ≤ 5/ > 5)	2.998	1.596–5.633	0.001**
Pathological stage	0.309	0.167–0.570	< 0.001***
Intrahepatic metastasis	2.942	0.867–9.984	0.084
STAG3 expression	0.114	0.048–0.272	< 0.001***
*Multivariate analysis*			
Age (y, ≤ 60/ > 60)	1.994	0.972–4.090	0.060
AFP (ng/mL, ≤ 20/ > 20)	1.000	0.516–1.940	1.000
Cirrhosis	1.398	0.670–2.916	0.372
Tumor size (cm, ≤ 5/ > 5)	1.025	0.511–2.055	0.944
Pathological stage	0.394	0.195–0.797	0.010*
STAG3 expression	0.174	0.063–0.483	0.001**

*CI* confidence interval, *HR* hazard ratio, *HCC* hepatocellular carcinoma.

*P < 0.05, **P < 0.01, ***P < 0.001.

### STAG3 overexpression suppresses the proliferation of HCC cells

**Fig. 2:**
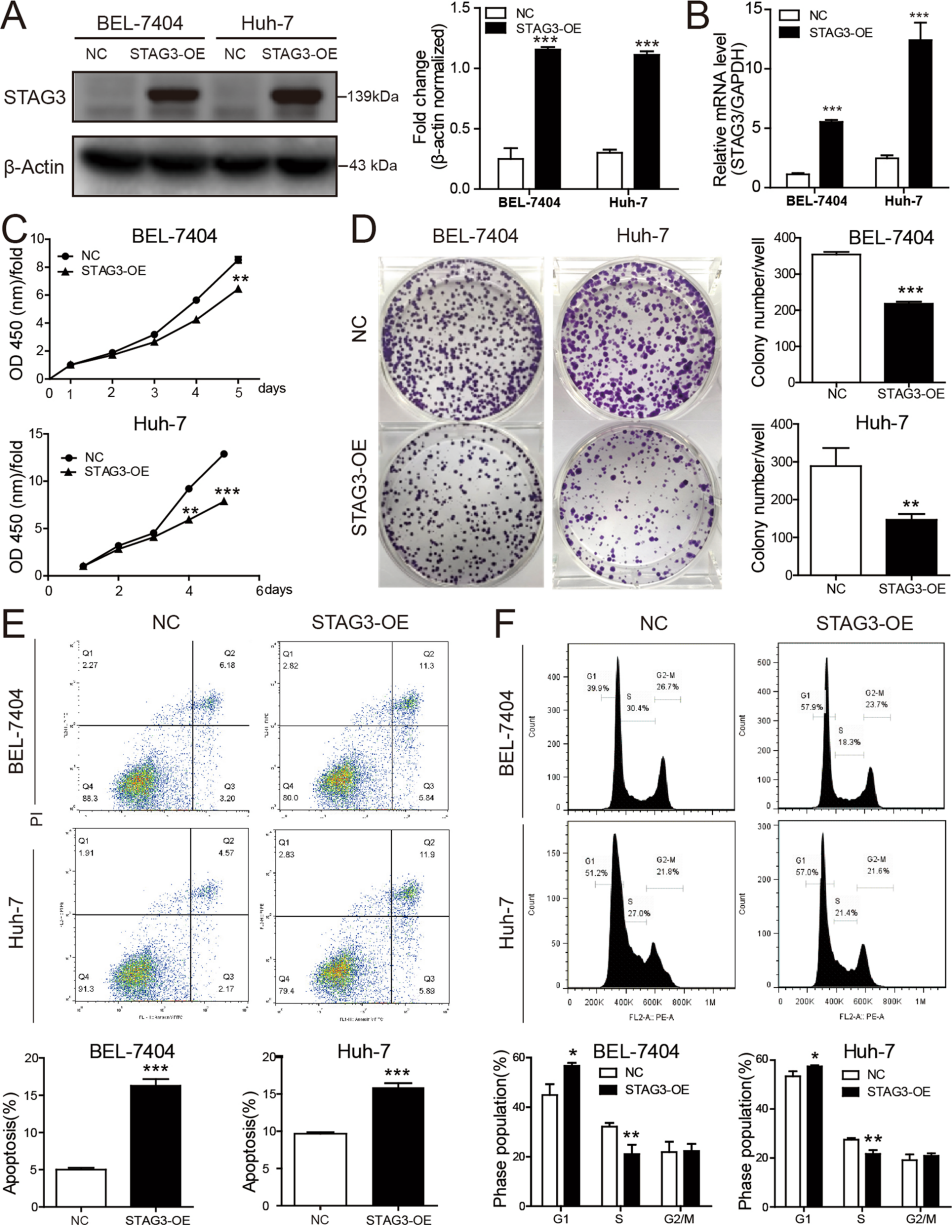
STAG3 overexpression inhibited cell proliferation, increased apoptosis and induced G1/S cell arrest in HCC. **A** The protein expression level of STAG3 in STAG3-OE HCC cells was detected by western blotting. **B** The mRNA expression level of STAG3 in STAG3-OE HCC cells was detected by RT–qPCR. **C** The effect of STAG3 overexpression on HCC cell proliferation was assessed by the CCK-8 assay. **D** The effect of STAG3 overexpression on HCC cell proliferation was assessed by a colony formation assay. The images represent colonies of cells belonging to the NC and STAG3-OE groups in six-well plates. **E** The apoptosis of STAG3-OE HCC cells was analyzed by flow cytometry. **F** The cell cycle of STAG3-OE HCC cells was analyzed by flow cytometry. *P < 0.05; **P < 0.01; ***P < 0.001

To gain insight into the function of STAG3, BEL-7404 and Huh-7 cells were infected with STAG3 overexpression lentivirus and its negative control. To ensure effective infection of cells in the NC group and STAG3 overexpression (STAG3-OE) group, the upregulation of STAG3 in HCC cells was demonstrated by western blot and RT–qPCR assays (Fig. 2A, B). Furthermore, cell proliferation was assessed by CCK-8 assays, and the observations suggested that STAG3 overexpression suppressed HCC cell proliferation (Fig. 2C) because the proliferation rate of STAG3-overexpressing cells was substantially lower than that of NC cells. Moreover, colony formation assays were performed to further determine the effect of STAG3 on cell proliferation, and the results (Fig. 2D) suggested that STAG3 overexpression resulted in a notable decrease in colony formation. The cells with unmodified expression of STAG3 exhibited higher proliferation rates than the STAG3-overexpressing cells.

### STAG3 overexpression promotes apoptosis and induces G1/S arrest in HCC cells

To explore the role of STAG3 in HCC cell apoptosis and the cell cycle, the effect of STAG3 on the apoptosis of BEL-7404 and Huh-7 cells was assessed by flow cytometry. We found that the STAG3-OE group exhibited a higher apoptosis rate than the NC group (Fig. 2E), and the STAG3-OE group had a higher percentage of G1-phase cells and a lower percentage of S-phase cells than the NC group (Fig. 2F). These results indicated that STAG3 overexpression promotes HCC cell apoptosis and G1/S cell arrest.

### STAG3 overexpression inhibits the migration and invasion of HCC cells

**Fig. 3:**
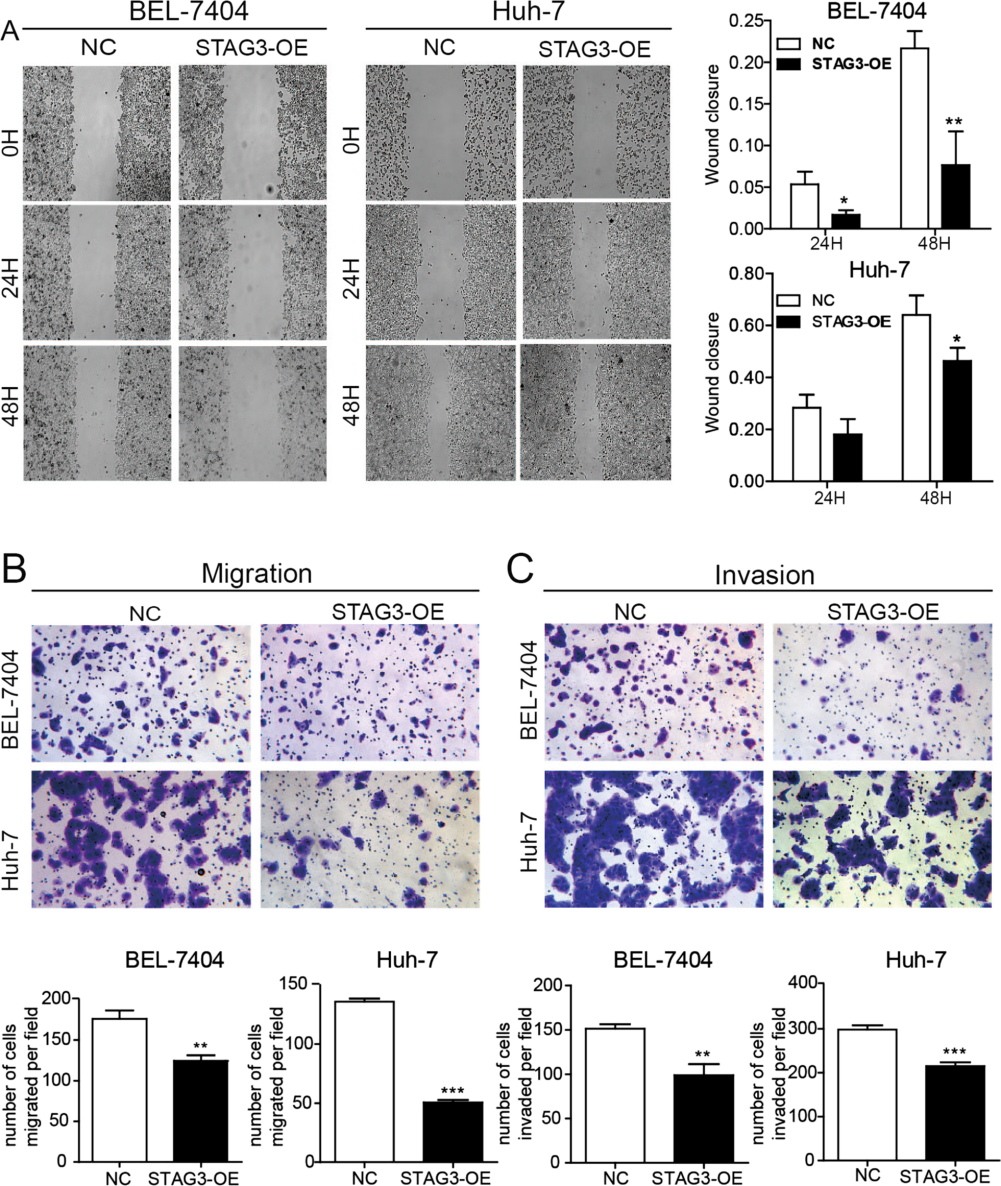
STAG3 overexpression inhibited HCC cell migration and invasion. **A** The distance traveled by HCC cells in the NC group and STAG3-OE group at 0 h, 24 h and 48 h after scratching was determined by the scratch wound healing assay. **B** The cell migration of HCC cells overexpressing STAG3 was detected by the Transwell migration assay. **C** The invasion of HCC cells overexpressing STAG3 was detected by the Transwell invasion assay. *P < 0.05; **P < 0.01; ***P < 0.001

To explore the role of STAG3 in HCC migration and invasion, STAG3 was overexpressed in BEL-7404 and Huh-7 cells. The distance of cell migration at a certain amount of time after scratching was evaluated to assess cell motility. The migration distance of STAG-OE cells was shorter than that of the corresponding NC cells (Fig. 3A). Transwell inserts were used to evaluate cell migration. The results showed that the migration of STAG-OE cells was significantly shorter than that of their corresponding NC cells (Fig. 3B). Similarly, the invasion of STAG-OE cells was significantly lower than that of their corresponding NC cells (Fig. 3C). These results indicated that STAG3 overexpression suppresses HCC cell migration and invasion.

### STAG3 overexpression suppresses the proliferation and induces the apoptosis of HCC in vivo

**Fig. 4:**
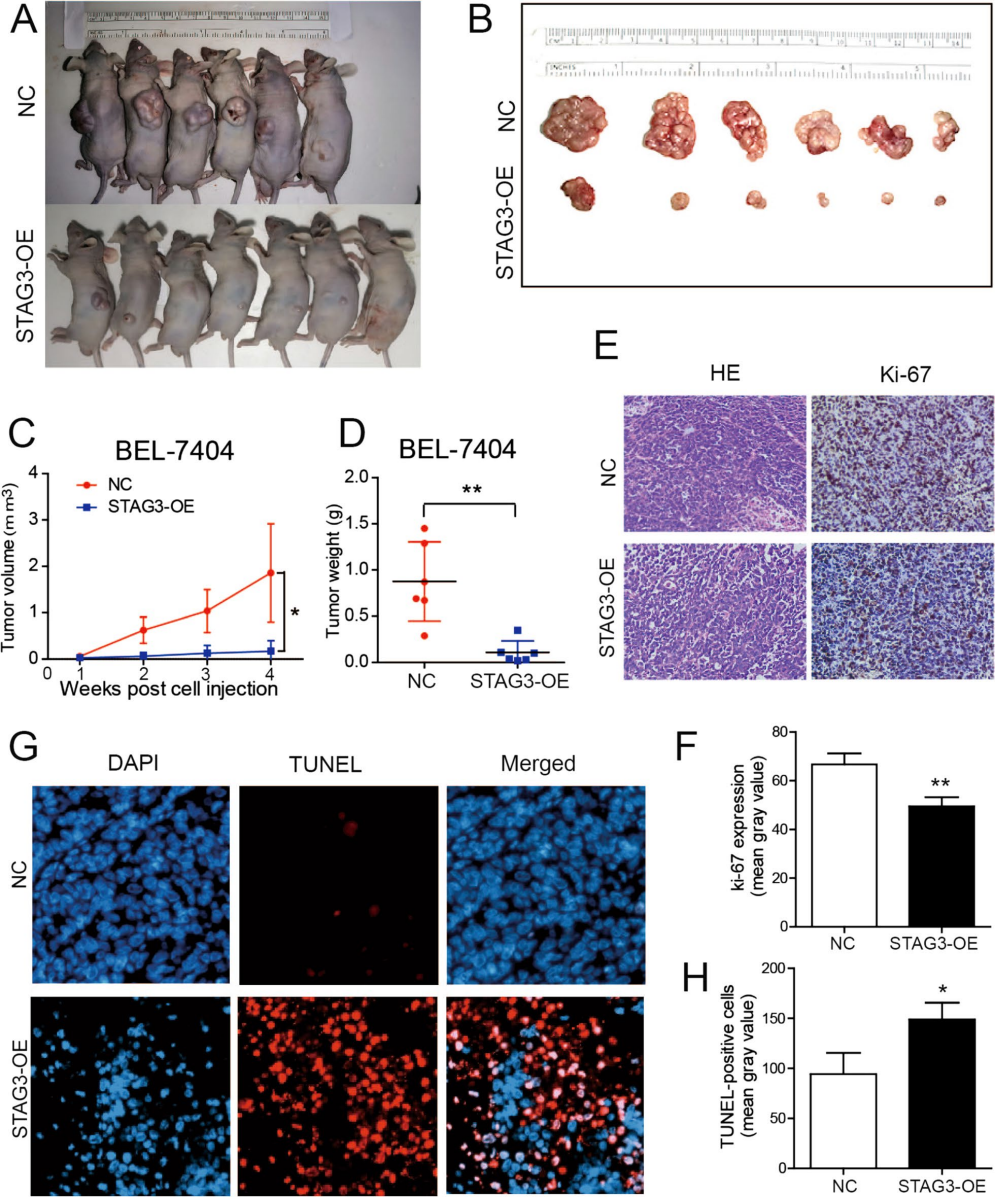
STAG3 overexpression inhibited HCC proliferation and promoted apoptosis in vivo. **A** The image shows mice with tumor xenografts inoculated with STAG3-OE BEL-7404 and STAG3-NC BEL-7404 cells. Xenograft tumor growth was monitored. **B** HCC tissues from mice with tumor xenografts inoculated with STAG3-OE BEL-7404 and STAG3-NC BEL-7404 cells. Representative mice and tumor images are included. **C** The category graph (symbols and lines) shows the results from the quantitative analysis of the HCC volume of the NC and STAG3-OE groups at 1–4 weeks. **D** The scatter plot (vertical) shows the results from the quantitative analysis of the HCC weight in the NC and STAG3-OE groups. **E** The expression of Ki-67 in xenograft tumor tissues from the NC and STAG3-OE groups was detected by IHC. Representative images of HE staining and IHC staining of Ki-67 are shown. **F** The percentages of Ki-67-positive cells are indicated. **G** A TUNEL assay was used to verify the apoptosis of xenograft tumor tissues from the NC and STAG3-OE groups. Representative images are shown. **H** The percentages of TUNEL-positive cells are indicated. *P < 0.05; **P < 0.01; ***P < 0.001

To verify the function of STAG3 in vivo, STAG3-OE BEL-7404 cells and normal BEL-7404 cells in the logarithmic growth phase were subcutaneously inoculated into the left back of nude mice. The volume and weight of tumors after STAG3-OE treatment were markedly lower than those of the tumors in the NC group (Fig. 4A–D), which indicated that STAG3 overexpression inhibited HCC cell proliferation. Furthermore, the results showed that the Ki-67-positive cell number was lower in mice engrafted with STAG3-OE cells (Fig. 4E, F). Moreover, the TUNEL staining results suggested that STAG3 overexpression promoted apoptosis in xenograft tumor tissue (Fig. 4G, H), and the results demonstrated that STAG3 effectively regulated tumor formation. Overall, we concluded that STAG3 overexpression suppressed the proliferation and induced the apoptosis of HCC in vivo.

### STAG3 may regulate biological functions via the Smad3-CDK4/6-cyclin D1 pathway and CXCR4/RhoA pathway in HCC cells

**Fig. 5:**
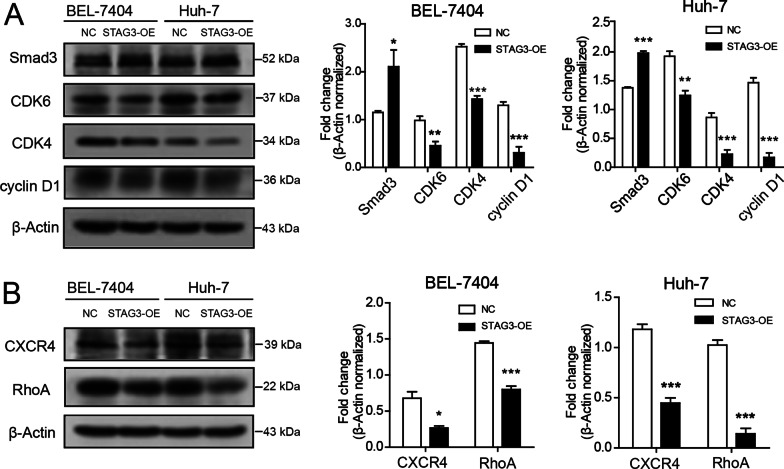
STAG3 regulates the Smad3-CDK4/6-cyclin D1 cell cycle pathway and CXCR4/RhoA pathway in HCC cells. **A** Western blot analysis of Smad3, CDK4, CDK6 and cyclin D1 in STAG3-OE HCC and NC HCC cells. β-Actin served as a loading control. **B** Western blot analysis of CXCR4 and RhoA in STAG3-OE HCC and NC HCC cells. β-Actin served as a loading control. *P < 0.05; **P < 0.01; ***P < 0.001

Cell cycle-related pathways play a crucial role in the tumor progression of distinct tumor types. To evaluate the influence of STAG3 expression on the Smad3-CDK4/6-cyclin D1 signaling pathway, important molecular proteins involved in the Smad3-CDK4/6-cyclin D1 signaling pathway were analyzed by western blotting. The results demonstrated that STAG3 overexpression increased the levels of CDK4, CDK6 and cyclin D1 in HCC cells and decreased the levels of Smad3 (Fig. 5A). Hence, STAG3 may regulate the Smad3-CDK4/6-cyclin D1 pathway in HCC cells. To explore other possibilities, the expression levels of some proteins specifically associated with cell cycle proteins, including CXCR4 and RhoA, were detected by western blot analysis. The outcomes revealed downregulated expression of CXCR4 and RhoA in HCC cells overexpressing STAG3 (Fig. 5B). Therefore, these results indicated that STAG3 might regulate biological functions through the Smad3-CDK4/6-cyclin D1 cell cycle signaling pathway and CXCR4/RhoA signaling pathway in HCC cells.

## Discussion

Liver cancer is the fourth leading cause of cancer-related death globally, and approximately 90% of primary liver cancer cases are classified as HCC [16]. Improvements in the diagnosis and treatment of HCC have been achieved, but the prognosis of HCC patients remains poor [17]. Therefore, there is an urgent need to understand the pathogenesis of HCC and provide new insights for its diagnosis and treatment. To explore the molecular mechanisms underlying HCC tumorigenesis and progression, we identified a novel HCC-correlated gene, STAG3, by analyzing a public TCGA dataset. STAG3 is a subunit of the specific adhesive protein complex that also regulates the cohesion of sister chromatids during cell division [18]. Knowledge of STAG3 is lacking, but some studies have shown that STAG3 is correlated with abnormalities, infertility and premature ovarian failure in women and have demonstrated the relationship between abnormal expression or mutation of STAG3 and male infertility and spermatogenesis disorders [19]. In this study, the expression, clinical significance, cellular function, and mechanism of action of STAG3 in HCC were further investigated.

STAG3 promotes the separation of chromosomes and the formation of sister chromatids by assembling and disassembling chromatin to facilitate the recombination and separation of homologous chromosomes [20] to ensure correct DNA repair and chromosome segregation [21]. When dysregulated, the STAG3 gene can block sister chromatid aggregation, and this blockage can facilitate the aggregation of sister chromatids, block the mutation of the STAG3 gene and cause chromosomal instability, which may potentially promote tumor progression [22]. In our study, STAG3 was found to be expressed at low levels in both HCC tissues and cell lines, as confirmed by molecular biology experiments. We subsequently found that low STAG3 expression was correlated with cirrhosis, higher AFP levels, larger tumor size and lower pathological stage. Moreover, low STAG3 expression was often associated with a poor OS rate in HCC patients, which was in accordance with the results from the analysis of TCGA data. These outcomes verified that STAG3 may play a crucial role in HCC tumorigenesis and progression. Furthermore, a multivariate Cox regression analysis showed that the expression of STAG3 combined with the pathological stage was an independent prognostic factor for OS in patients with HCC. These results suggest that STAG3 could be a promising prognostic biomarker for HCC patients. Moreover, to explore the roles of STAG3 in HCC tumorigenesis and progression, a series of cell functional experiments were conducted. The outcomes suggested that the overexpression of STAG3 inhibited proliferation, migration and invasion, promoted apoptosis of HCC cell lines, inhibited proliferation and promoted apoptosis in vivo. Overall, the results indicate that STAG3 may be used as a potential target for molecular therapy in HCC.

Smad proteins can be posttranslationally modified by a variety of proteins and can interact with a variety of transcriptional activators/repressors with a wide range of biological functions. Smad proteins can be posttranslationally modified by a variety of proteins and can interact with a variety of transcriptional activators/repressors with a wide range of biological functions [23, 24]. TGF-β type I receptor (TβRI) and c-Jun N-terminal kinase (JNK) differentially phosphorylate the mediator Smad3 to yield two distinct phosphoisoforms: C-terminally phosphorylated Smad3 (pSmad3C) and linker-phosphorylated Smad3 (pSmad3L). In mature hepatocytes, oncogenic signaling via the JNK/pSmad3L pathway antagonizes signaling via the tumor-suppressive TβRI/pSmad3C pathway. The Smad phosphoisoforms may represent important biomarkers for predicting HCC in nonalcoholic steatohepatitis (NASH) [25]. Therapies targeting the downstream signaling mediated by Smad3 and cyclin D1, such as CDK4/6 inhibitors, could provide new therapeutic options for the treatment of antiandrogen-resistant disease [26]. Cyclin D, CDKs, CDK inhibitors and tumor suppressor proteins control the initiation and termination of the cell cycle, and imbalances in this process can thus lead to uncontrolled cell proliferation and ultimately cancer [27]. The cyclin D1-CDK4/6 complex initiates or regulates the early G1 process via the phosphorylation of downstream Rb, and excessive phospho-acidification of Rb attenuates cell cycle inhibition to lead to uncontrolled cell proliferation [28, 29]. These cell cycle proteins are known to play a crucial role in the cell cycle, and STAG3 encoding for a meiosis-specific protein that is also involved in the cell cycle. We hope to study the mechanism from the perspective of the cell cycle. In our study, we found that STAG3 overexpression upregulated Smad3 expression and downregulated CDK4, CDK6, and cyclin D1 expression.

CXCR4 is a seven transmembrane G protein-coupled receptor for CXCL12 (also called SDF-1) and a central receptor in tumor biology, and its expression controls proliferation and tumor cell survival in various tumor types [30, 31]. Some researchers have suggested that CXCR4-overexpressing cells downregulate the expression of cyclin D1 and Bcl-2 and activate other signaling pathways such as MAPK to support cell proliferation and tumor progression [32]. Furthermore, the CXCR4 antagonist induces the apoptosis of acute myeloid leukemia (AML) cells, and this apoptosis is mediated by the upregulation of miR-15a/miR-16-1, which results in the downregulation of the target genes cyclin D1 and Bcl-2 [33]. Studies have shown that CXCR4 overexpression activates cyclin D1 expression and that CXCR4 silencing reduces the cyclin D1 expression levels [34]. Moreover, the CXCR4/RhoA pathway is involved in tumor progression in digestive tract tumors [35]. RhoA is a family of GTPases, and several studies have revealed high RhoA expression in various cancers and described the active role of RhoA in different pathways that are implicated in tumorigenesis. Furthermore, suppression of the Rho pathway has been shown to improve outcomes in different malignant tumors, such as hepatocellular, lung and gastric cancers [36]. The initial studies focused on the regulation of intracellular skeletal proteins by RhoA [37], and subsequent studies demonstrated that RhoA activation could upregulate the expression of cyclin D1 and downregulate the expression of cyclin D1 kinase inhibitors [38]. Therefore, CXCR4 and RhoA can directly or indirectly regulate cell cycle processes, and STAG3 is closely related to cell division. We hope to study the molecular mechanism of STAG3 in HCC from the perspective of the cell cycle. In this study, a western blot analysis showed that STAG3 overexpression downregulated the expression of CXCR4, RhoA and cyclin D1. These results suggest that STAG3 may regulate the biological functions of HCC cells through the Smad3-CDK4/6-cyclin D1 cell cycle pathway and CXCR4/RhoA pathway. However, the molecular mechanism through which STAG3 plays a role needs to be further studied in HCC.

**Fig. 6:**
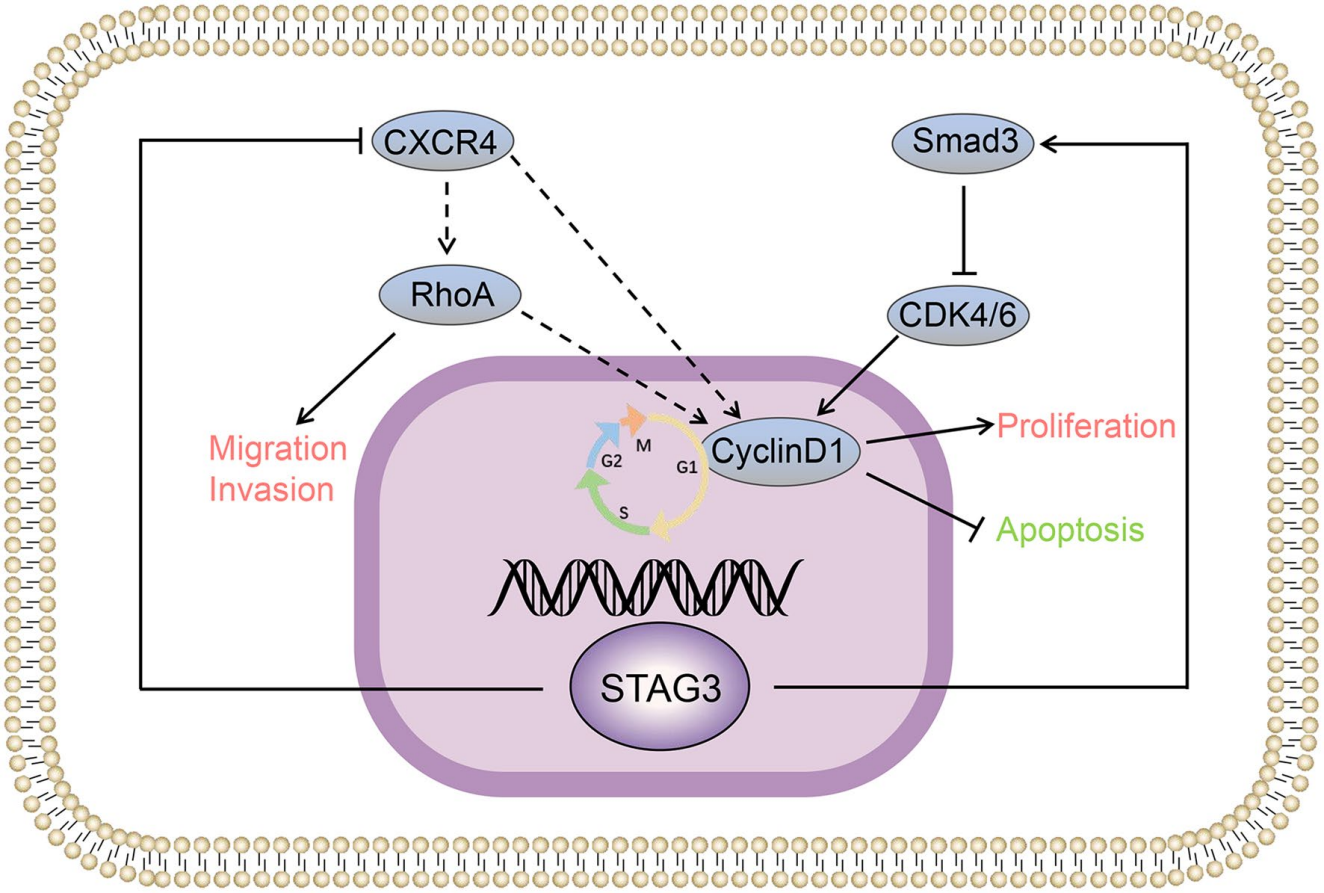
STAG3 regulates the biological functions of HCC through the Smad3-CDK4/6-cyclin D1 pathway and CXCR4/RhoA pathway. Pictorial representation of the outcome of our study

In conclusion, we conducted cell biology and animal experiments and found that STAG3 could inhibit cell proliferation, promote cell apoptosis, and induce G1/S cell cycle arrest in HCC. STAG3 also inhibited the migration and invasion ability of HCC cells and was found to play a tumor suppressive role in HCC. The mechanism through which STAG3 regulates the biological functions of HCC was investigated, and the results indicate that STAG3 may play a role through the Smad3-CDK4/6-cyclin D1 pathway and CXCR4/RhoA pathway (Fig. 6). STAG3 may be a tumor suppressor gene in human HCC, and obtaining a more in-depth understanding of its function may lead to the discovery of a promising therapeutic target for improving the clinical treatment of HCC.

## Conclusions

STAG3 overexpression suppresses the biological behaviors of HCC by regulating the Smad3-CDK4/CDK6-cyclin D1 and CXCR4/RhoA pathways. Therefore, STAG3 is a tumor suppressor gene with the potential to become a new therapeutic target in HCC.

## Supplementary Information

**Additional file 1.** Fig. S1 STAG3 downregulation is frequently observed in HCC patients.

**Additional file 2** Original Data of Western Blot.

## Data Availability

The data generated or analyzed during this study are available from the corresponding author upon reasonable request (https://figshare.com/s/f7b918421d6bed84c89c).
