# Supplementary material for: The upregulation of stromal antigen 3 expression suppresses the phenotypic hallmarks of hepatocellular carcinoma through the Smad3-CDK4/CDK6-cyclin D1 and CXCR4/RhoA pathways
Source: BMC Gastroenterol. 2022 Aug 8;22:378. doi: 10.1186/s12876-022-02400-z (PMC9361574; doi:10.1186/s12876-022-02400-z)
Supplement: Supplementary file 1 — Additional file 1. Fig. S1 STAG3 downregulation is frequently observed in HCC patients. [file 12876_2022_2400_MOESM1_ESM.docx]

Upregulated stromal antigen 3 suppresses the biological behaviors of hepatocellular carcinoma by regulating the cell cycle pathway

Menglin Zhao^a,1^, Yanyan Wang^a,1^, Yue Zhang^a^, Xinwei Li^a^, Jiaqi Mi^a^, Qiang Wang^b^, Zhijun Geng^c^, Lugen Zuo^d^, Xue Song^c^, Sitang Ge^d^, Zining Zhang^e^, Mingyue Tang^a^, Huiyuan Li^a^, Zishu Wang^a^, Chenchen Jiang^f,*^, Fang Su^a,*^

^a^Department of Medical Oncology, The First Affiliated Hospital of Bengbu Medical College, No. 287 Changhuai Road, Bengbu, Anhui 233030, China

^b^Department of Network Information Center, Bengbu Medical College, No. 2600 Donghai Road, Bengbu, Anhui 233030, China

^c^Department of Central Laboratory, The First Affiliated Hospital of Bengbu Medical College, Bengbu, China

^d^Department of Gastrointestinal Surgery, The First Affiliated Hospital of Bengbu Medical College, No. 287 Changhuai Road, Bengbu, Anhui 233030, China

^e^Department of Clinical Medicine Science, Bengbu Medical College, No. 2600 Donghai Road, Bengbu, Anhui 233030, China

^f^Cancer Neurobiology Group, School of Medicine & Public Health, The University of Newcastle, Callaghan NSW 2308, Australia

^1^These authors contributed equally to this work and share the first authorship.

^*^Correspondence to: Fang Su, MD, PhD. The First Affiliated Hospital of Bengbu Medical College, No. 287 Changhuai Road, Bengbu, Anhui 233030, China. Email: [sufang2899@163.com;](mailto:sufang2899@163.com;) sufang@bbmc.edu.cn. Tel: +86-0552-308116. Chenchen Jiang, PhD. The University of Newcastle, Callaghan NSW 2308, Australia. Email: chenchen.jiang@newcastle.edu.au. Tel: +61-2-49217233.

**Supplementary Material**

**Figure S1**

**
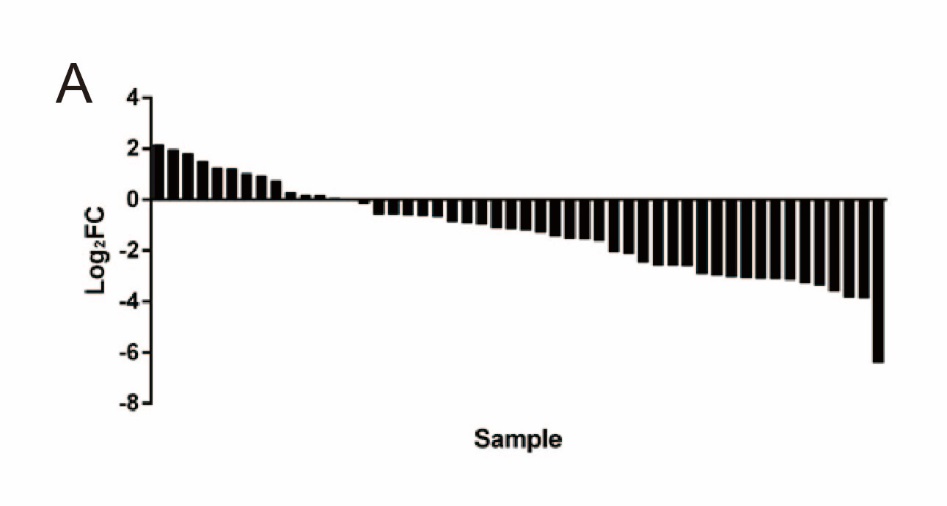
**

**Fig. S1** STAG3 downregulation is frequently observed in HCC patients. (A) Total of 50 paired samples were collected from HCC patients. Compared with normal liver tissues, STAG3 expression upregulated in 27 patients with HCC, STAG3 expression downregulated in 7 patients, and STAG3 expression unchanged in 16 patients. STAG3, stromal antigen 3; HCC, hepatocellular carcinoma.
