# Supplementary figures and images for: The upregulation of stromal antigen 3 expression suppresses the phenotypic hallmarks of hepatocellular carcinoma through the Smad3-CDK4/CDK6-cyclin D1 and CXCR4/RhoA pathways
Source: BMC Gastroenterol. 2022 Aug 8;22:378. doi: 10.1186/s12876-022-02400-z (PMC9361574; doi:10.1186/s12876-022-02400-z)

Figure 2A

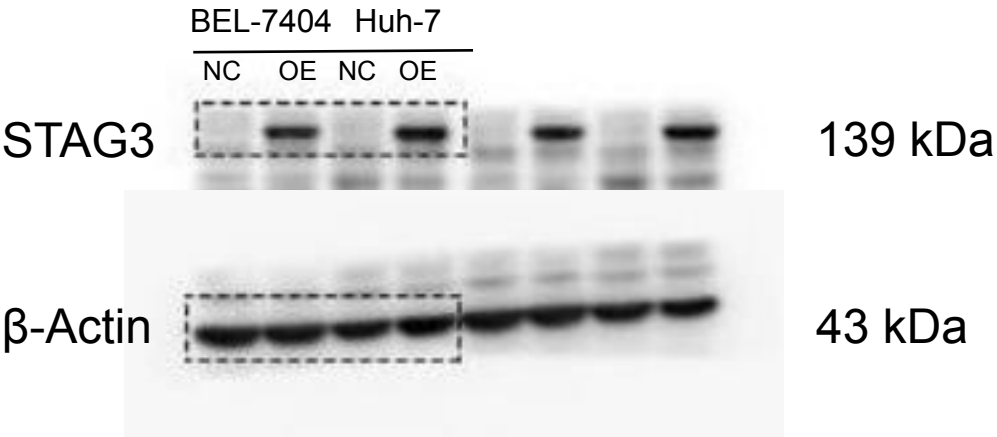

Figure 5B

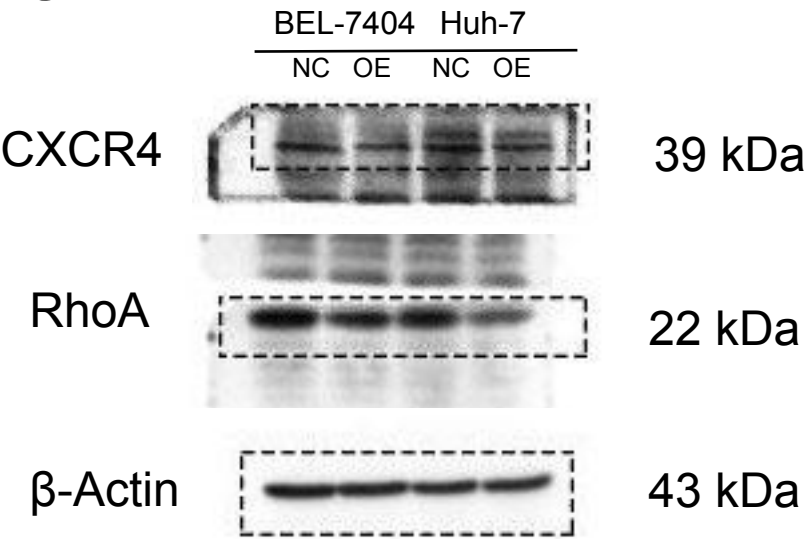

Figure 5A

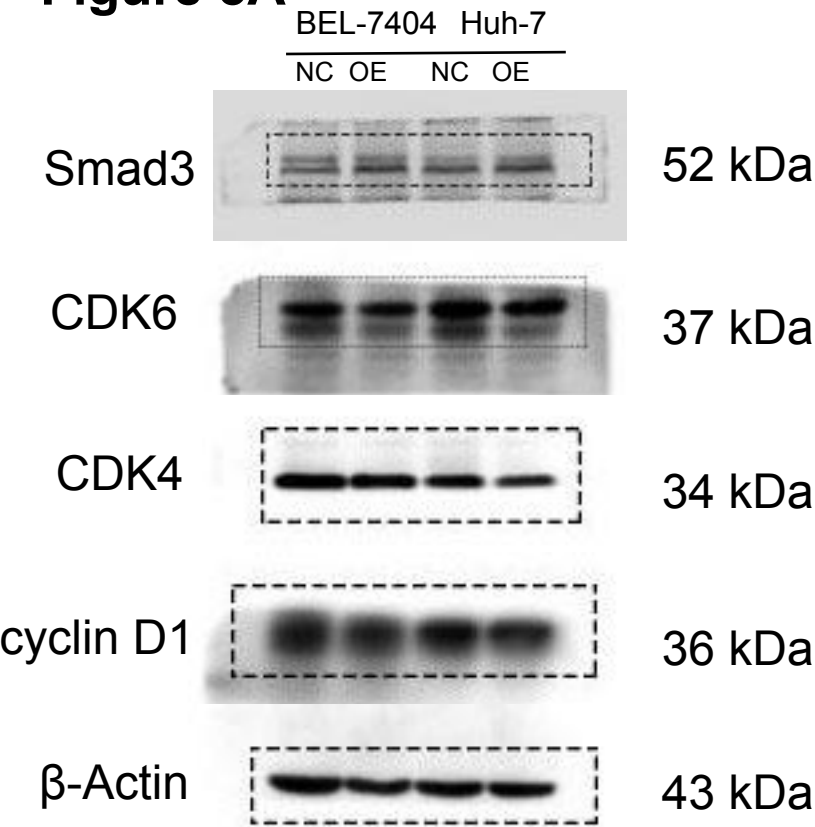

Figure 2A

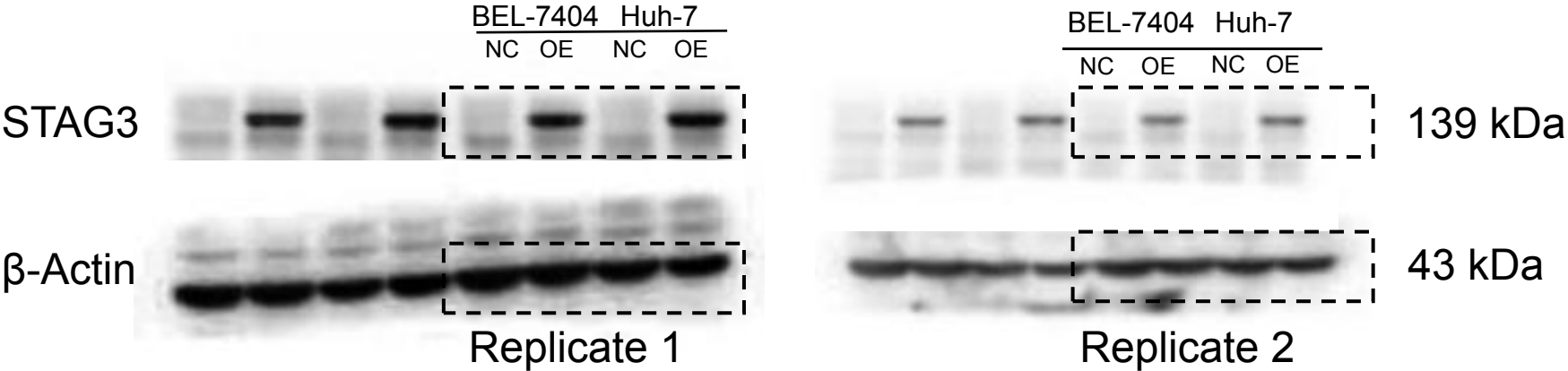

Figure 5A

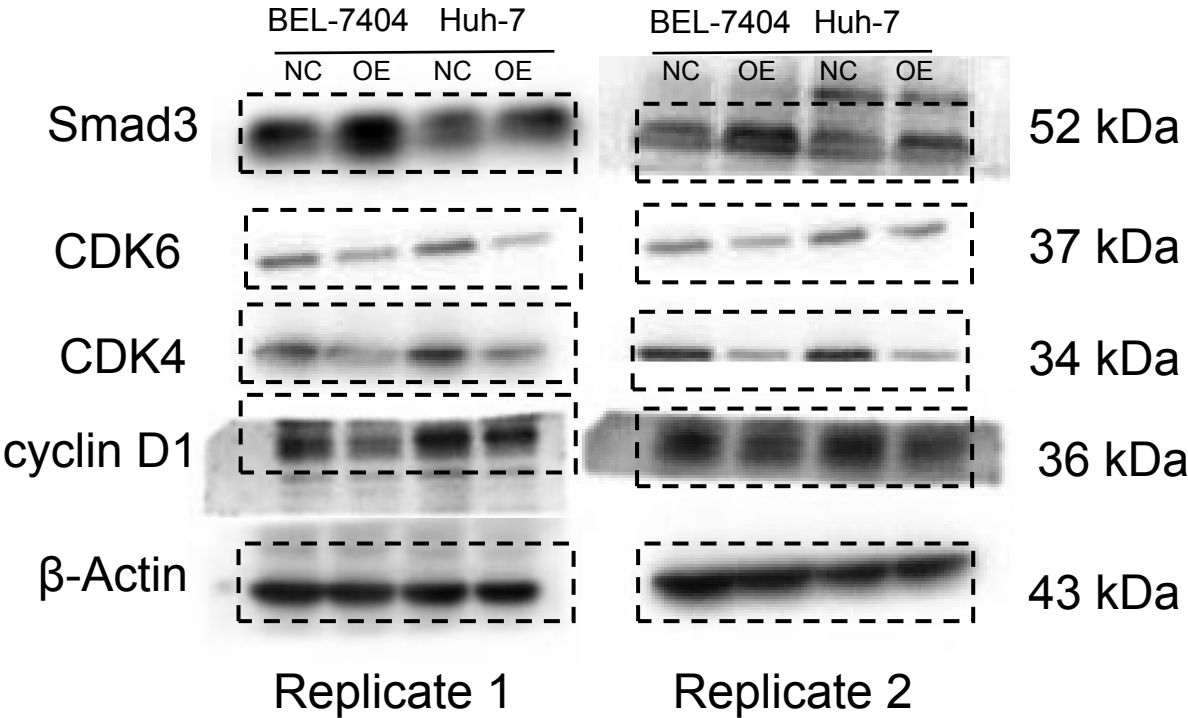

Figure 5B

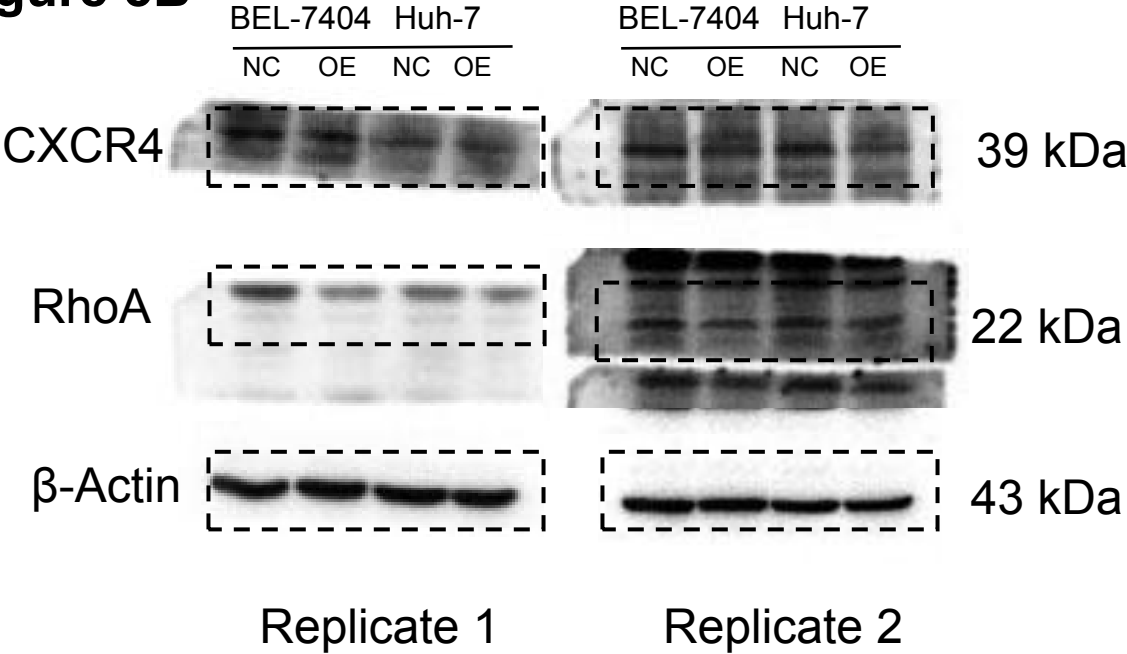

Supplement: Supplementary file 2 — Additional file 2 Original Data of Western Blot. [file 12876_2022_2400_MOESM2_ESM.pdf]
